# A generalized logistic-logit function and its application to multi-layer perceptron and neuron segmentation

**DOI:** 10.3389/frai.2026.1785867

**Published:** 2026-06-09

**Authors:** Wenqi Gu, Yingtao Zhang, Alessandro Muscoloni, Carlo Vittorio Cannistraci

**Affiliations:** 1 Center for Complex Network Intelligence (CCNI), Tsinghua Laboratory of Brain and Intelligence (THBI), Department of Psychological and Cognitive Sciences, Tsinghua University, Beijing, China; 2 School of Biomedical Engineering, Tsinghua University, Beijing, China; 3 Department of Computer Science and Technology, Tsinghua University, Beijing, China

**Keywords:** generalized logistic-logit function, input feature modulator, multi-layer perceptron, neural network, neuron segmentation

## Abstract

Logistic and logit functions play important roles in modern science, serving as foundational tools in various applications, such as artificial neural networks (ANN). While there are functions that could produce distinct logistic and logit curves, no single, unified framework has been developed to generate both logistic and logit curves. We introduce a Cannistraci–Muscoloni–Gu generalized logistic–logit function (CMG-GLLF) to fill this gap. CMG-GLLF provides four interpretable and trainable parameters that allow explicit control over: curve type and steepness, asymmetry, and upper and lower limits of x- and y-axes. CMG-GLLF’s potential is explored in basic machine intelligence tasks. As a proof-of-concept on how this function can improve the performance of deep learning, we propose a trainable input feature modulator (IFM) that consists of learning the parameters of the CMG-GLLF for each input layer node during backpropagation for a multi-layer perceptron (MLP), which is a fundamental building block of many complex network architectures. Compared to various other learnable functions, across three different optimizers, CMG-GLLF allows superior MLP’s accuracy and stable training behavior on CIFAR-10 and CIFAR-100 image classification, but at the cost of increased computational time. Hence, we identified limitations to address in future studies, notably the need to derive an explicit mathematical expression for the logit phase, which could: (i) mitigate numerical instability in more complex architectures (e.g., CNNs) while reducing computational overhead and (ii) enable a systematic evaluation of CMG as an activation function across all layers. Furthermore, CMG-GLLF, adopted as a data transformation function, enhances the accuracy of affinity-graph-based neuron segmentation. CMG-GLLF combines in a unique framework the ability of logistic and logit functions to modulate signals or variables, covering a full spectrum of attenuation or amplification transformations. CMG-GLLF is flexible and trainable, has the potential to advance machine learning models, and can inspire further applications in other data analysis challenges in different domains of science.

## 1 Introduction

Logistic and logit functions play pivotal roles in various fields such as economics, medicine, and computer science (Boateng and Abaye, 2019; Dubey et al., 2022; Kwasnicki, 2013; Ramos, 2013). The logistic function is a fundamental mathematical tool widely employed across diverse fields due to its unique S-shaped curve and bounded output. In statistics and machine learning, it forms the core of logistic regression, enabling effective modeling of binary outcomes and probabilistic predictions (Cramer, 2003; Hosmer et al., 2013). In biology, the logistic function is used to describe growth phenomena under resource constraints, capturing the transition from exponential growth to saturation (Marciniak-Czochra, 2003; Wu et al., 2020). Its smooth, differentiable nature also makes it indispensable in artificial neural networks, where it serves as a non-linear activation function, facilitating gradient-based learning (Dubey et al., 2022). Meanwhile, the logit function, the inverse of the logistic function, maps probabilities from the interval (0,1) onto the entire real line by transforming a probability 𝑝 into its log-odds. This transformation is central to logistic regression: by modeling the log-odds in terms of predictors, the logit function allows one to capture how the linear combination of covariates and predictors affects the probability of an event happening (Hosmer et al., 2013). The versatility of the logistic and logit functions in modeling growth, decision-making, classification, and intelligent systems underscores their enduring significance in both theoretical and applied research.

Nevertheless, the standard logistic and logit functions are expressed as follows:


$$\text{logistic}(x)=\frac{1}{1+e^{-x}}$$

$$\text{logit}(x)=\ln(\frac{x}{1-x})$$

It has limited flexibility, which restricts its ability to capture complex real-world phenomena—particularly in cases of imbalanced, skewed, or asymmetric growth and response behaviors. To overcome these shortcomings, researchers have introduced generalized logistic (Richards curve) and generalized logit functions with additional tunable parameters, thereby extending their adaptability to a wider range of scientific applications (Prasetyo et al., 2020; Richards, 1959) (See Supplementary material 1 for a detailed description). Yet, these approaches still face important limitations:

1. Lack of exact boundaries: Generalized logistic and logit functions do not provide exact reachable lower and upper bounds on either the x-axis or y-axis, posing difficulties in applications that require strict input/output boundaries and precise mappings. For example, when modeling the relationship between project time *t* and completion rate r: at the start (*t* = 0), *r* = 0%, and values below this have no practical meaning; at the deadline (*t* = T), *r* = 100%, and values beyond this point are impossible.
2. Limited shape control: The steepness and asymmetry of generalized logit functions are determined only by the relative relationship between two parameters instead of using separate parameters that independently control these curve characteristics.

To address these issues, we introduced the Cannistraci–Muscoloni-Gu generalized logistic–logit function (CMG-GLLF, denoted as CMG below for simplicity), which not only fills these gaps but also provides the first unified framework for generating both generalized logistic and logit curves. Derived from the generalized logistic function (Richards, 1959), CMG offers the following:

1. Explicit control over exact reachable lower and upper bounds on both x and y axes
2. Independent control of steepness and asymmetry through inflection rate and deviate inflection point.
3. An approximation algorithm to derive the logit curve by inverting the generalized logistic curve, introducing more flexibility into the logit curve.
4. A unifying inflection rate parameter that enables smooth transitions between step functions, logistic functions, linear functions, and constant functions.

We explored CMG’s potential in machine learning through two main applications. First, CMG can be adopted as an input feature modulator (IFM), assigning each input feature a CMG curve with learnable parameters (via gradient update in back propagation) to enhance deep learning performance, with negligible parameter increase. Unfortunately, meticulously testing the CMG on large neural network architectures would require a large budget that we do not have available; therefore, instead of performing a few unreliable tests on many large network architectures, we opted for a proof-of-concept to conduct a deep and extensive study on a multi-layer perceptron (MLP). This is because the MLP is a fundamental building block of many artificial neural network structures. However, to design a challenging stress-test that could help us to investigate the advantage of CMG properly, we selected non-trivial tasks for MLP, which are CIFAR-10 and CIFAR-100 image classification. This could allow us to fairly investigate the superiority of CMG as IFM compared to no-IFM direct input and many other learnable functions.

In addition to performance, practical aspects such as numerical stability, computational overhead, and their variations across different neural network architectures are important when introducing new functional modules into neural networks. In particular, the implicit approximation procedure required for the logit-phase of CMG may introduce additional computational cost and raise concerns about training stability. Therefore, we included a systematic evaluation of the numerical stability and computational overhead of CMG as IFM in MLP under three different optimizers, and we added a preliminary analysis on a simple convolutional neural network (CNN).

CMG as IFM increased the performance of MLP while retaining numerical stability, but at the cost of increased computational time. Hence, we also identified limitations to address in future studies, most notably the need to derive an explicit mathematical expression for the logit phase. Such an expression could (i) mitigate numerical instability in more complex architectures such as CNNs while reducing computational overhead, and (ii) enable a systematic evaluation of CMG as an activation function across all layers.

Second, we demonstrate that CMG can improve the accuracy of an affinity-graph-based neuron segmentation algorithm by transforming affinity graphs with CMG mappings (Funke et al., 2019). Finally, since CMG is interpretable and explainable, we analyzed the type of signal transformation that results from CMG application.

Overall, CMG provides a powerful new family of input feature modulators and data transformation tools for machine learning. With its high flexibility, optimized CMG curves can be tailored to diverse tasks; hence, they might be investigated in future studies for accelerating the deployment of machine learning models in both research and industry.

## 2 Results and discussion

### 2.1 Cannistraci–Muscoloni–Gu

The expression of CMG is given by


$$\mathrm{CMG}(x)=\{\begin{array}{c} y_{L}+\frac{y_{R}-y_{L}}{1+\frac{x_{\max}-x}{x-x_{\min}}e^{\left(2-\frac{1}{\mu })(\frac{x-x_{\min}}{x_{\max}-x_{\min}}-I\right)}},\text{if}\;0\leq \mu \leq 0.5\;\\ f^{-1}(x,1-\mu ),\text{if}\;0.5<\mu \leq 1 \end{array}$$

**Figure 1:**
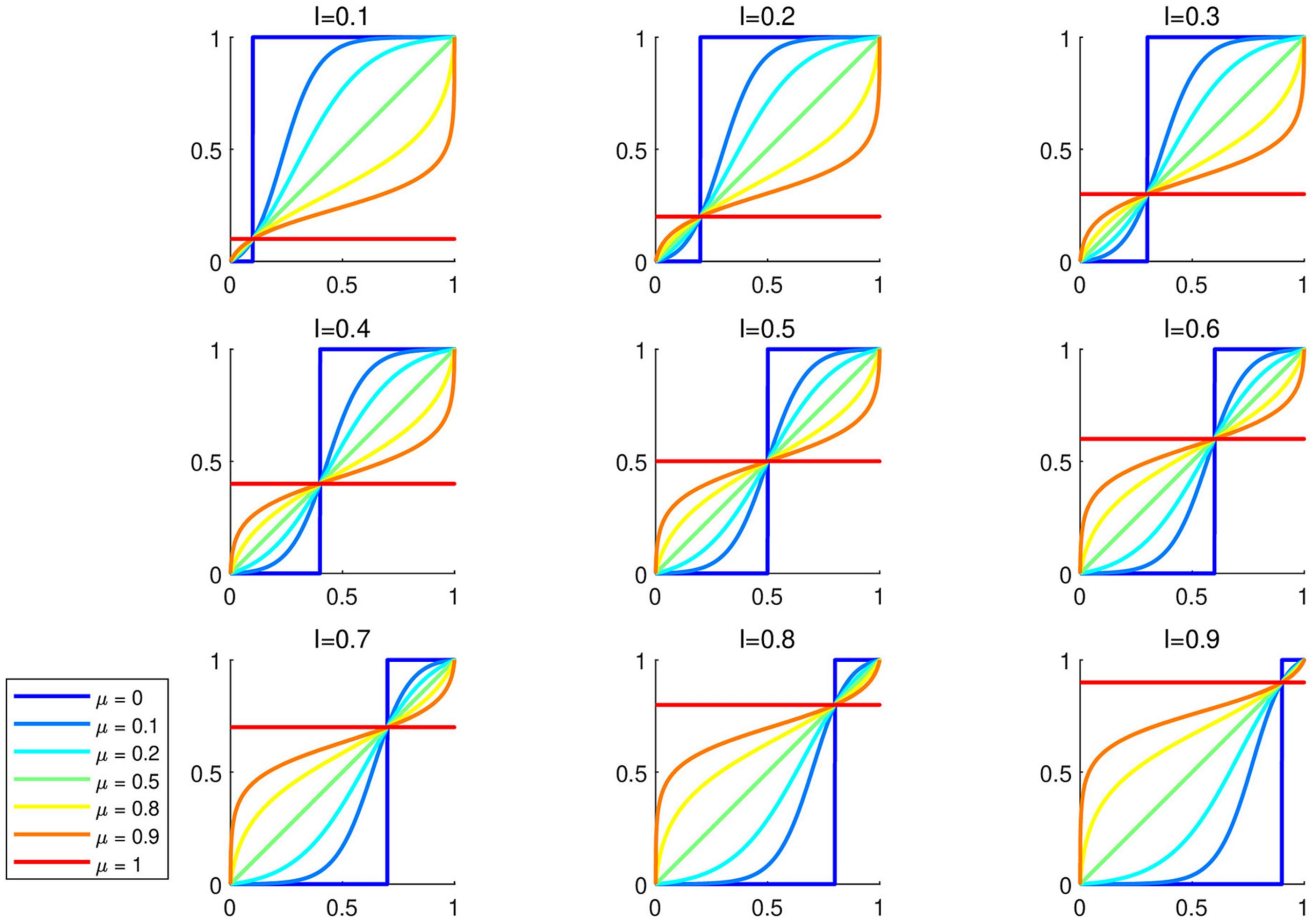
CMG curve. It shows how the inflection parameter *μ* and deviate inflection point I affect the shape of CMG curve. When μ=0 , CMG is a step function, and the discontinuity happens at the inflection point; when 0<μ<0.5 , it is a generalized logistic function; lower μ results in a steeper curve; when μ=0.5 , CMG becomes a linear function; when 0.5<μ<1 , it is a generalized logit function (inverse logistic function), and the higher the μ is the more gradual the curve becomes; when μ=1 , function values remain constant at the inflection value. I determines the asymmetry of the curve, specifically at what proportion of x range the deviate inflection point occurs. In this figure, other parameters are set as: $x_{\min}=y_{L}=0$ , $x_{\max}=y_{R}=1.$

The text below and Figure 1 explain how different parameters affect the shape of the CMG curve.

-
$x_{\min}$ and $x_{\max}$ are the minimum and maximum values of x, respectively.
-
$y_{L}$ and $y_{R}$ are the corresponding y values of $x_{\min}$ and $x_{\max}$
-
$x_{I}\;$ is deviate inflection point determining the asymmetry of the curve, and I controls the relative position of it on the x-axis, specifically

$$x_{I}=x_{\min}+I.(x_{\max}-x_{\min})$$

- when 0<I<0.5 , the curve is left-skewed
- when I=0.5 , the curve is symmetric about the deviate inflection point
- when 0.5<I<1 , the curve is right-skewed
-
μ is the inflection rate controlling the steepness and the type of CMG curve, specifically:
- When μ=0 , the curve is a step function

$$\mathrm{CMG}(x)=\{\begin{array}{c} y_{L},\text{if}\;x<x_{I}\\ y_{R},\text{if}\;x>x_{I}\\ \max(y_{L},y_{R}),\text{if}\;x=x_{I} \end{array}$$

- When 0 < *μ* < 0.5, it is a logistic curve, and a lower μ results in a steeper curve
- When μ = 0.5, it is a linear curve

$$\mathrm{CMG}(x)=y_{L}+(x-x_{\min}).\frac{y_{R}-y_{L}}{x_{\max}-x_{\min}}$$

- When 0.5 < μ < 1, it is a logit curve derived from an approximation algorithm that inverts the logistic curve, and a lower μ results in a steeper curve.
- When μ = 1, it becomes a constant function defined as

$$\mathrm{CMG}(x)=y_{I}=y_{L}+I.(y_{R}-y_{L})$$

Here, for simplicity and brevity, we only report the function, and we comment on its formulation. For readers interested in the detailed theoretical derivation of CMG and the approximation algorithm, please refer to Supplementary material 2.

### 2.2 CMG as MLP neural network input feature modulator (IFM)

The goal of this section is to offer evidence that CMG is trainable. To this aim, we demonstrate that by applying CMG as an input feature modulator for MLP in image classification, the accuracy and learning speed can be greatly improved. The motivation to design an IFM is the hypothesis that task-oriented learning of the input feature value distribution during end-to-end training provides a better representation of input data to improve the model’s performance. Specifically, we assign each input feature a learnable CMG curve during end-to-end training to modulate (amplifying or attenuating) the feature values. During training, *μ* and I can be learned within the limit of the definition range (0 < μ < 1, 0 < I < 1). This means that the MLP has 2*Ni parameters more to train, where Ni is the number of nodes in the input layer. The upper and lower limits of the x- and y-axes are data specific, in which the lower bound for x and y values are minimal in the input data batch, and the upper bound is the maximal value in the input batch. This is to ensure only the value distribution is reshaped, but the original feature range is kept invariant.

We focus on MLP for image classification rather than on more recent and complex neural network architectures because MLP represents one of the simplest network forms and is therefore well-suited to evaluate the net effect of the IFM, avoiding interference from other performance-enhancing mechanisms, such as convolutional layers (Lecun et al., 1998; Rumelhart et al., 1986). In addition, we conducted an extensive set of experiments to evaluate multiple CMG training scenarios and to compare them with other transformation functions; therefore, we opted for the MLP to moderate GPU computing costs.

**Figure 2:**
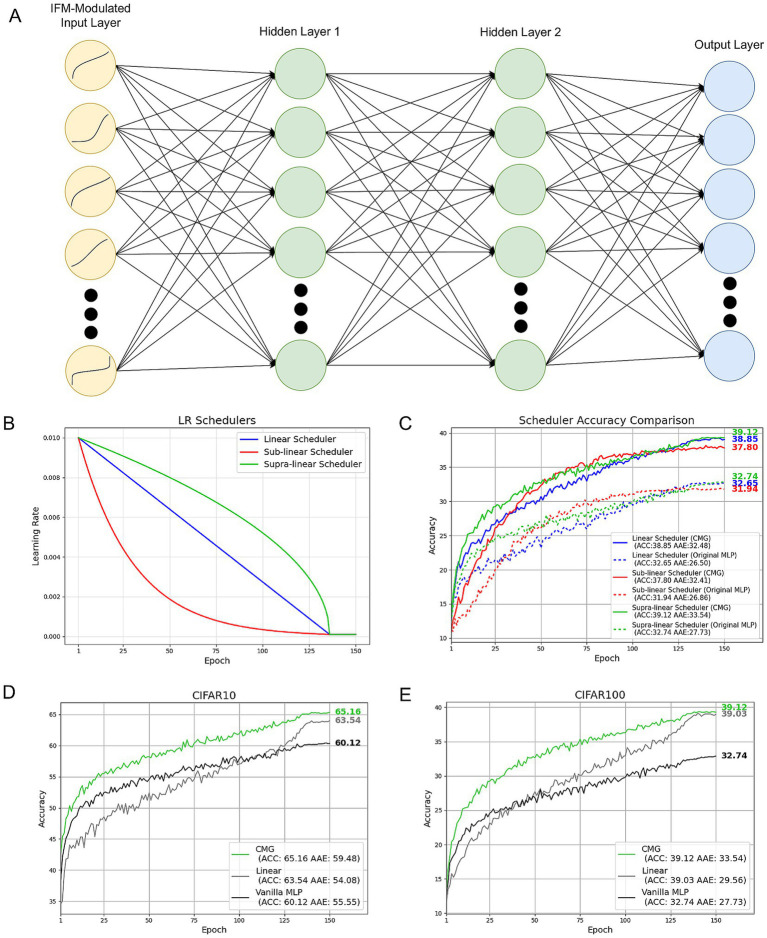
CMG is an input feature modulator for MLP on SGD. **(A)** Illustration of MLP where the features at the input layer are modulated by CMG **(B)**. Comparison of different schedulers’ learning rate decay **(C)**. Evaluation results of different schedulers. In the legend, each trial’s accuracy (ACC) and area across the epochs (AAE) are shown. The accuracy of each trial is also shown beside the curve, and this is the same for **(D,E) (D)** Test accuracy curves of MLP modulated by CMG, linear (best-performance counterpart learnable function), and vanilla MLP on CIFAR10 **(E)** Test accuracy curves of MLP modulated by CMG, linear (best-performance counterpart learnable function), and vanilla MLP on CIFAR100.

We trained the CMG on the MLP as a stress test to cope with two image classification tasks that are difficult for a basic neural network architecture: CIFAR-10 (in which the task is to classify a colored 32×32 image into one of the 10 object categories) and CIFAR-100 (similar to CIFAR-10, but there are 100 object categories, which is more challenging) (Krizhevsky, 2009). For CIFAR-10, we adopted an MLP with two hidden layers with size {1,024,512} as performed by Wesselink et al. (2024). This means that for CIFAR-10, the IFM costs a 0.17% (2*Ni/total number of parameters = 2*3072/ ((3,072 * 1024) + 1,024 + (1,024 * 512) + 512 + (512 * 10) + 10 + 2*3072)) parameter increase. For CIFAR-100, we extended the hidden layer dimension by 2 to enable the network to deal with a more complex task (Wesselink et al., 2024). This means that for CIFAR-100, the IFM costs a 0.07% (2*Ni/total number of parameters = 2*3072/ ((3,072 * 2048) + 2048 + (2048 * 1024) + 1,024 + (1,024 * 100) + 100 + 2*3072)) parameter increase. Figure 2A depicts the MLP with an input feature modulator in the input layer. We also extended the number of training epochs in (Wesselink et al., 2024) from 100 to 150 for a deeper investigation of IFM’s potential. The training configurations are described in Section 4.3.

In the experiments, the highest accuracy in all epochs is taken to measure the classification performance, and the area across epochs (AAE) proposed in Zhang et al. (2024) is utilized to measure the learning speed (calculation detailed in Section 4.6).

In ANN training, a learning rate scheduler is widely applied to gradually decay the learning rate as training proceeds, which enables a smooth transition from exploration at the beginning and exploitation in the end. We investigated three learning rate (LR) schedulers with different decay patterns to determine which one works best on MLPs with CMG input feature modulator (CMG) and without IFM (vanilla MLP). We trained MLPs using an SGD optimizer, which is one of the most popular optimizers in deep learning. Linear scheduler decays linearly, while sub-linear scheduler decays faster at first and then gradually reduces the decay speed, and supra-linear scheduler has the inverse tendency of sub-linear scheduler. After training for 90% of total epochs, the learning rate for 3 schedulers converges at the same point and remains the same for the rest of the training. The detailed formulas for the schedulers are provided in the Methods section. Figure 2B depicts the learning rate decay of three schedulers in 150 epochs with $LR_{\text{init}}=0.01$ . We evaluated these three schedulers on CIFAR100 (the most complicated task in the study) to determine which works best, and the results shown in Figure 2C reveal that the supra-linear scheduler has the best accuracy and AAE in both CMG and vanilla MLP. Thus, a supra-linear scheduler is employed in all the following experiments. Furthermore, to show the performance boost brought by CMG is not simply from increasing the number of parameters in the network, we tested four other learnable functions with two tunable parameters as its counterpart for a fair comparison (Detailed in Supplementary material 4) using the SGD optimizer, and CMG was the best performing. Not still satisfied, to foster a deeper and complete research, we tested whether CMG can outperform learnable functions with more than two parameters, comparing CMG against a 4-parameter learnable function SReLU (Jin et al., 2015) (Detailed in Supplementary material 5).

Figures 2D,E show the accuracy of MLP modulated by CMG, linear transformation (best-performance counterpart function), and vanilla MLP on test sets across the epochs for CIFAR10 and CIFAR100, respectively. As can be seen here across both datasets:

- By adopting CMG as IFM, we can greatly improve both the accuracy and learning speed compared to the vanilla MLP. On CIFAR10 (Figure 2D) and CIFAR100 (Figure 2E), we achieve an absolute improvement of 5.04 and 6.38% compared to vanilla MLP. Also, adopting CMG can increase AAE for 3.93 and 5.81% on CIFAR10 and CIFAR100, respectively.
- Among the five different two-parameter IFM functions, CMG achieves both the best accuracy and highest learning speed, followed by the linear transformation function (Supplementary material 4).
- Compared against SReLU, a four-parameter IFM function, CMG has a higher learning speed on both datasets, and it achieves superior accuracy on CIFAR-10 and comparable accuracy on CIFAR-100 (Supplementary material 5).

**Figure 3:**
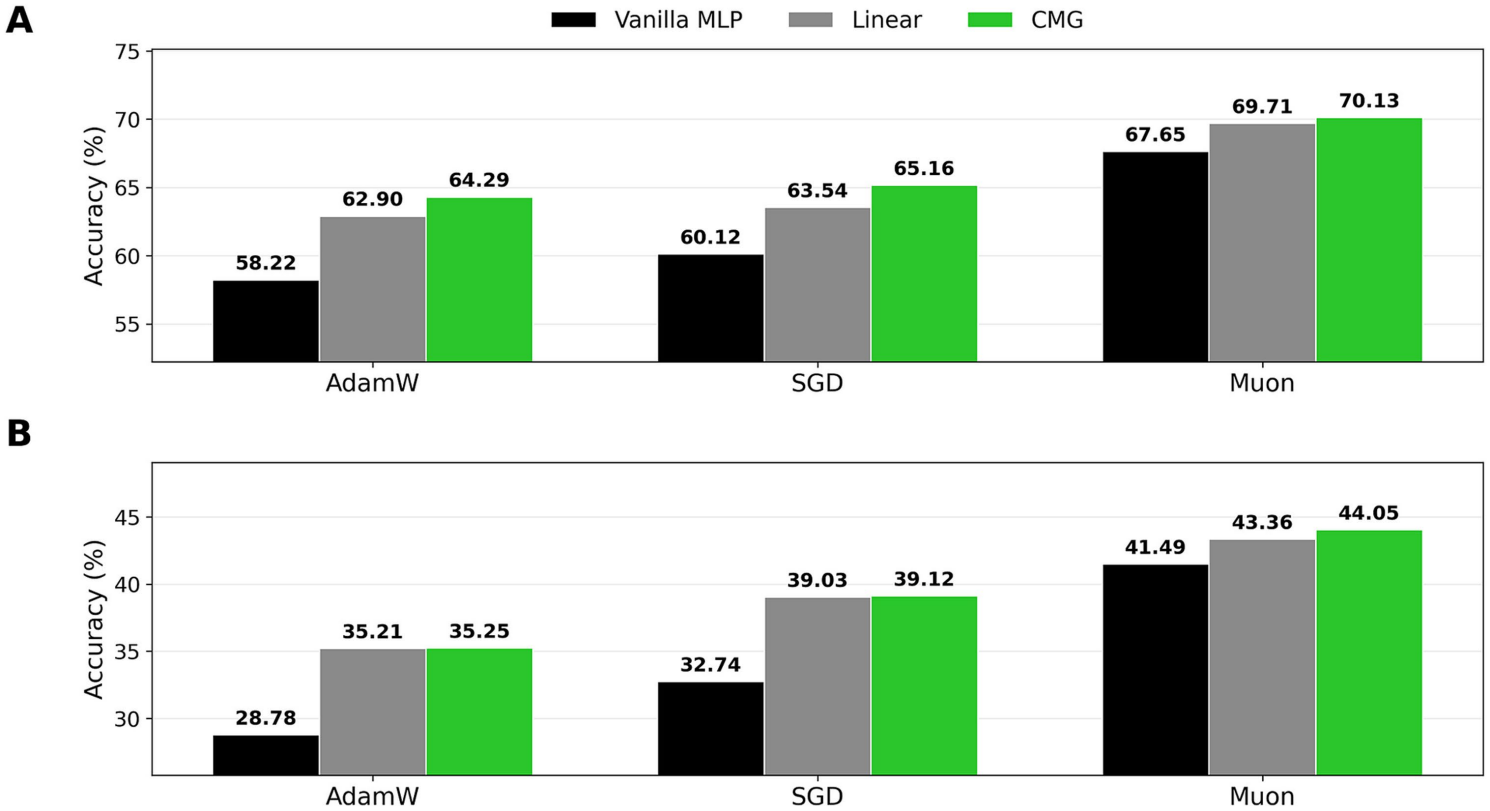
Test accuracy of vanilla MLP, linear and CMG on different optimizers. **(A)** Accuracy results of different methods on CIFAR10. **(B)** Accuracy results of different methods on CIFAR100.

To show IFM’s numerical stability during training, we train vanilla MLP, linear, and CMG using another two optimizers, AdamW and recently-proposed Muon, which has demonstrated strong empirical performance and improved convergence in training modern neural networks (Jordan, 2024; Loshchilov and Hutter, 2019). The test accuracies are shown in Figure 3. It shows that on whichever optimizer, CMG can consistently outperform linear IFM and vanilla MLP on both CIFAR10 and CIFAR100. Supplementary Table 1 records the occurrence of events including non-finite loss (NF Loss), non-finite gradient (NF Grad), and non-finite parameters (NF Param) during training. It shows that in all experiments, there is no occurrence of unstable training events, indicating that training is stable regardless of the optimizer used when using CMG as IFM.

Considering training CMG as IFM requires using an approximation algorithm to calculate the logit-phase CMG values in the forward pass, we also evaluated the computational overhead, including peak GPU memory usage and training time. As shown in Supplementary Table 2, CMG requires more GPU memory and training time compared to linear IFM and vanilla MLP. This is the current limitation of CMG as IFM, and it will be commented in the Discussion section.

**Figure 4:**
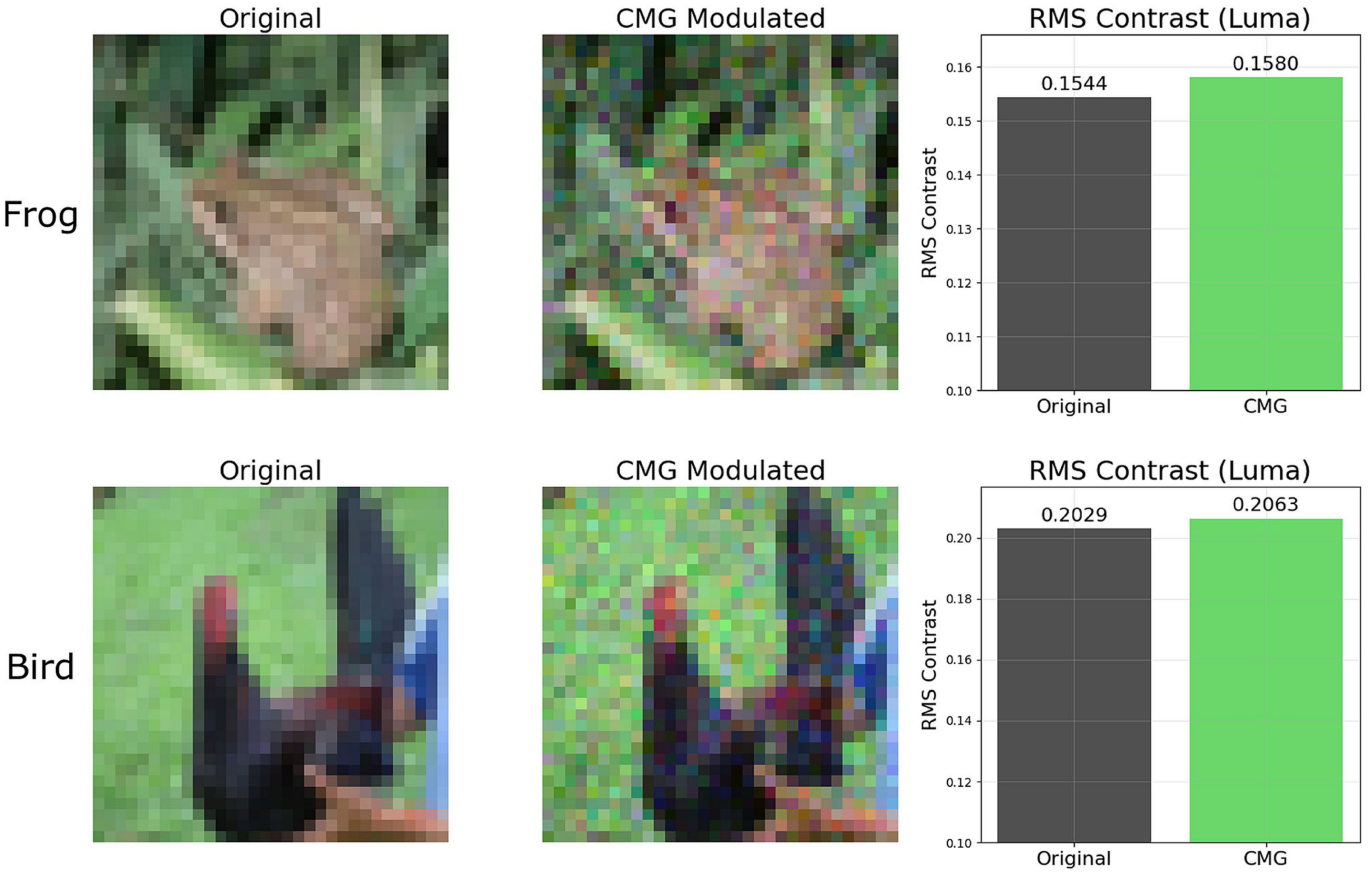
Comparison between images before and after CMG modulation. (Leftmost) The original image in the CIFAR10 test set (middle), image after CMG modulation (right). Bar plot comparing the RMS contrast between the original image and the CMG-modulated image.

Furthermore, to gain a better understanding of how CMG improves the learning on MLP, we visualize the test set image of CIFAR10 before and after CMG modulation in Figure 4, where the learned IFM with the best accuracy is adopted. Taking the categories “frog” and “bird” as examples, it could be observed that after CMG modulation, there is a clearer brightness contrast in the image. For example, the important features such as the eye of the frog and the crest of the bird are more highlighted after modulation. To quantitatively validate this phenomenon, for each image, luma (Guidance for operational practices in HDR television production, 2022) (a proxy for measuring brightness, the higher the brighter) of each pixel is calculated. By calculating the root mean square (RMS) of lumas in the image, the extent of brightness contrast can be measured (Peli, 1990). The RMS Contrast (Luma) bar plot in Figure 4 shows that the brightness of pixels is more dispersed after CMG modulation, indicating IFM provides a stronger brightness contrast after modulation. The methods to calculate luma and RMS are described in Section 4.5.

### 2.3 Preliminary results of CMG as input element modulator in CNN

We also include a preliminary analysis of CMG working as an element modulator of the input channels of a CNN architecture with three convolutional layers and one fully-connected layer to investigate how CMG might change behavior when applied to more complex structures. This means for each element in the input image to CNN, we assigned a trainable CMG curve as a modulator. Supplementary Figure 3 shows the test accuracy on the CIFAR10 and CIFAR100 datasets, illustrating that CMG trained within the CNN framework has lower accuracy than vanilla CNN. In Supplementary Table 3, the numerical stability results are analyzed, showing that instability occurs during training for CMG. Interestingly, the performance of the linear IFM is higher than that of the vanilla CNN, and it does not display any numerical instability during training. These results point out that while the idea of IFM is valid in general, the CMG solution needs adjustment that should be investigated in future studies in order to be applied to more complex architectures than MLP.

### 2.4 Improving affinity-graph-based neuron image segmentation algorithm with CMG

**Figure 5:**
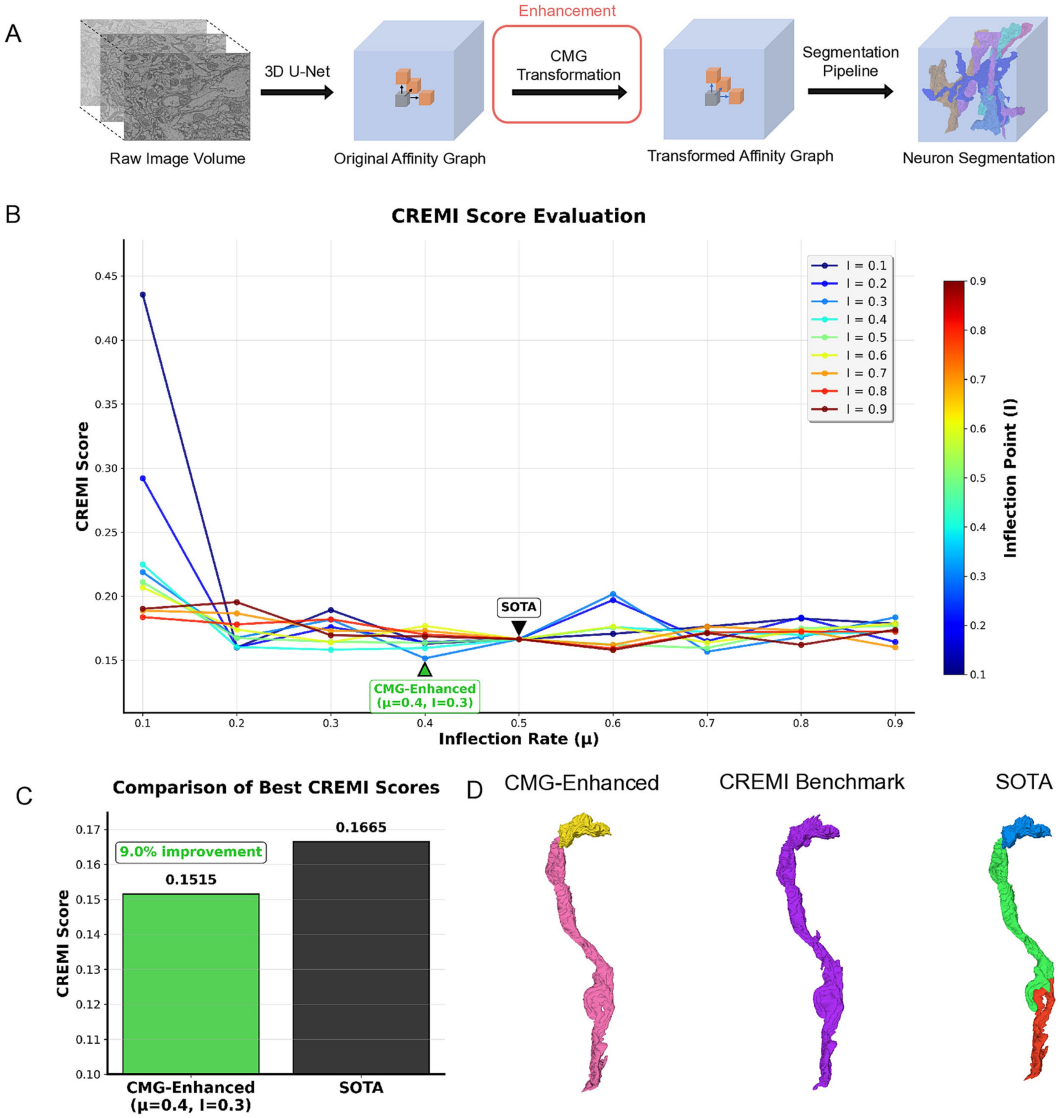
CMG transformation improves the EM image neuron segmentation algorithm. **(A)** The working pipeline of the neuron segmentation experiment. First, the 3D brain EM image volume is sent into a 3D U-Net (Çiçek et al., 2016), which predicts the affinity values (black arrows between smaller cubes) between neighboring voxels in the volume. Affinity values in the original affinity graph (middle left) are then transformed by the CMG function for enhancement, and the new values are indicated by blue arrows in the transformed affinity graph (middle right). Finally, the transformed affinity graphs are segmented using the pipeline proposed by Funke et al. (2019) to produce the predicted neuron segments (rightmost). **(B)** CREMI score (the lower the better) evaluation on CREMI training set B. Each line represents one inflection point (I) and the *x*-axis shows the μ values. SOTA (black dot) and best CMG-enhanced (μ = 0.4 and I = 0.3) are highlighted with black and green arrows, respectively. **(C)** Bar plot comparing the CREMI score (the lower the better) between the best result obtained using CMG-enhanced (μ = 0.4 and I = 0.3) and SOTA. **(D)** Segmentation quality comparison between CMG-enhanced, CREMI benchmark, and SOTA.

In this section, we demonstrated the effectiveness of CMG in improving the accuracy of an algorithm that segments neurons in brain electron microscopy (EM) images. Here, we utilized the re-aligned, augmented Drosophila brain EM 3D image from the CREMI challenge as the benchmark dataset and the CREMI score as the evaluation metric [detailed in Section 4.6; (Funke et al., 2016)]. The segmentation pipeline proposed by Funke et al. (2019) is adopted as the SOTA method (see Figure 5A). Specifically, the pipeline first predicts an affinity graph for the 3D brain image, which estimates the probability that each pair of adjacent voxels in the image belongs to the same neuron segment (referred to as the original affinity graph here). We can enhance the sharpness of the affinity graph weights by increasing their contrast with the CMG that acts as a soft thresholding transformation. Then, an aggregation algorithm merges the voxels into neuron segments based on the affinity graph weight values, which involves two segmentation parameters: the merge function (MF) and a thresholding parameter.

Here, the motivation to apply CMG as a data transformation tool is to adjust the weight value distribution in the original affinity graph, resulting in transformed affinity graphs, aiming to improve the accuracy of the final segmentation. A grid search is performed on a range of different CMG parameters to determine the optimal combination of inflection rate (*μ*) and inflection point (I) for enhancing segmentation performance. The upper and lower limits are data-specific and are fixed at the minimal and maximal affinity values in the affinity graph to reshape only the distribution of values, but keep the affinity value range invariant. For each affinity graph, including both the transformed and the original, a grid-search involving five different merge functions and nine thresholds is applied to produce the segmentations. Table 1 shows the grid-search space for neuron segmentation experiments. For both the original and transformed affinity graph, the segmentation that yields the lowest CREMI score is used to represent the performance of the state-of-the-art (SOTA) and CMG-enhanced SOTA (denoted as CMG-enhanced here), respectively.

**Table 1:** Grid-search space for neuron segmentation experiments.

Parameter	Value
Inflection rates (μ)	0.1–0.9 in increments of 0.1
Deviate inflection points (I)	0.1–0.9 in increments of 0.1
Merge functions (MF)	{median_aff_histograms, 85_aff_histograms, median_aff, 85_aff, max_10}
Thresholds	0.1–0.9 in increments of 0.1

Compared to the SOTA method proposed by Funke et al. (2019), CMG-enhanced shows a clear improvement in neuron segmentation quality. As shown in Figures 5B,C, when μ = 0.4 and I = 0.3, the segmentation result produced using the transformed affinity graph achieves a CREMI score that is 9.0% lower than that of the original affinity graph.

Additionally, we conducted a qualitative evaluation of neuron segmentations produced with different methods (Figure 5D). In the middle is a neuron segment in the CREMI benchmark. Its corresponding neuron segments in CMG-enhanced segmentation are made of two segments, with the pink one preserving most of the structures in the benchmark. While those in SOTA segmentation are more fractured, it splits the neuron segments into three parts, causing more split errors.

## 3 Discussion

We developed the Cannistraci–Muscoloni–Gu generalized logistic–logit function (CMG-GLLF), the first unified framework capable of generating both logistic and logit curves. Its tunable parameters enable flexible control over curve type, steepness, asymmetry, and the upper and lower limits on both the x- and y-axes.

By assigning each feature a learnable CMG curve as input feature modulator (IFM) for MLP during backpropagation, we greatly improved the performance of fully connected MLPs with a negligible parameter increase (0.17% in CIFAR-10 and 0.07% in CIFAR-100), achieving superior accuracy and stable training across three different optimizers, AdamW, SGD, and Muon. Moreover, the CMG curve enhanced the accuracy of a neuron image segmentation algorithm by transforming its intermediate product—the affinity graph. These experiments demonstrate that CMG, as a simple yet flexible computational tool, holds promise for advancing machine learning models.

Despite these advantages, CMG has certain limitations. First, interpreting the deviate inflection point in CMG presents challenges. In classical logistic curves, the inflection points mark the location of the maximum growth rate. However, because CMG introduces the parameter Q to control bounds on both axes, the inflection point becomes deviated, which means it aligns with the maximum growth rate only when I = 0.5. Although our definition of the deviate inflection point remains a useful reference for curve behavior, it introduces deviations that should be carefully considered when applying CMG to specific tasks where the position of maximum growth rate is extremely important.

Second, while CMG showed to be robust to numerical instability as IFM on MLP architecture regardless of the adopted optimizer, it requires more GPU memory and training time compared to vanilla MLP. Furthermore, evidence of numerical instability appeared on more complicated architectures, such as CNNs. These issues are associated with the approximation algorithm to invert the logistic curve that requires the use of the implicit function theorem to calculate approximated gradients, which leads to computational overhead and risks of numerical instability during backpropagation. To address this relevant issue, we conducted a new study that is currently available as a pre-print by Gu and Cannistraci (2026) in which we present the derivation of a new explicit, fully differentiable CMG expression. We derive this by approximating the inverse of the logistic phase using Newton’s method with one iteration step. The results of the application of the explicit CMG on the MLP and CNN adopted in the current article show that the issues of numerical instability and computational overhead are addressed, with CMG performing as the best IFM not only on this CNN but also on VGG-16 (Simonyan and Zisserman, 2015). We further managed to adopt CMG as hidden layer activation functions on both classical MLP and physics-informed neural networks (PINN; (Raissi et al., 2019)), improving the performance in PINN by a magnitude compared to the benchmark activation function, illustrating the wider applicability of CMG in the field of deep learning. Furthermore, the fact that CMG parameters can be learned during training allows us to use the distribution of their values as a new tool for explainability of MLP and PINN (Gu and Cannistraci, 2026).

In future studies, CMG’s potential should be investigated on more diverse deep learning architectures and applications. Leveraging CMG as a learnable activation function in neural networks could become a key factor for their explainability.

## 4 Materials and methods

### 4.1 Datasets

Here, CIFAR-10 and CIFAR-100 datasets are adopted as benchmark image classification datasets for evaluating the performance of the input feature modulator (Krizhevsky, 2009). Both of them consist of 32 × 32 colored images. CIFAR-10 contains 60,000 images evenly distributed across 10 classes, while CIFAR-100 contains the same number of images but divided into 100 classes with 600 images per class. Each dataset is split into 50,000 training and 10,000 test images.

For neuron segmentation, a publicly available *Drosophila melanogaster* brain electron microscopy (EM) image dataset from MICCAI 2016 CREMI challenge is adopted here to show the effectiveness of CMG in improving the accuracy of the automatic neuron segmentation algorithm (Funke et al., 2019). Our experiment utilizes the re-aligned augmented CREMI training set B provided in Funke et al. (2019) as the benchmark. The ground-truth labeling provided is an instance segmentation of neurons in 3D images, where voxels sharing the same neuron label belong to the same neuron segment.

The code for reproducing the results in this article can be found on GitHub: https://github.com/biomedical-cybernetics/CMG-GLLF.git

### 4.2 Learning rate schedulers

For a deeper investigation of how different learning rate decay patterns can impact the training of MLP with input feature modulator, we designed three different learning rate schedulers, in which the linear scheduler decays linearly, while the sub-linear scheduler decays faster at first and then gradually reduces the decay speed, and the supra-linear scheduler has the inverse tendency of the sub-linear scheduler. Their expressions are detailed below:

Denoting $LR_{\text{current}}$ as the current learning rate, $LR_{\text{init}}$ as initial learning rate, T as total number of epochs, and t as current epoch,

- Linear scheduler

$$\mathrm{LR}(t)=\{\begin{array}{c} LR_{\text{init}}*(0.01+0.99*(1-\frac{t}{0.9*T}))\;\text{if}\;0\leq t\leq 0.9*T\;\\ LR_{\text{init}}*0.01\;\;\text{if}\;0.9*T<t\leq T \end{array}$$

- Sub-linear scheduler

$$\mathrm{LR}(t)=\{\begin{array}{c} LR_{\text{init}}*{\left(0.01\right)}^{\frac{t}{0.9*T}}\;\;\;\;\text{if}\;0\leq t\leq 0.9*T\;\\ LR_{\text{init}}*0.01\;\;\text{if}\;0.9*T<t\leq T \end{array}$$

Supra-linear scheduler


$$\mathrm{LR}(t)=\{\begin{array}{c} LR_{\text{init}}*(0.01+0.99*\sqrt{1-\frac{t}{0.9*T}})\;\text{if}\;0\leq t\leq 0.9*T\;\\ LR_{\text{init}}*0.01\;\;\text{if}\;0.9*T<t\leq T \end{array}$$

### 4.3 IFM training setups

Here, to evaluate the performance of IFM, we adopted MLP to train on two popular benchmark image classification tasks: CIFAR-10 and CIFAR-100. To analyze the performance and stability of CMG on different optimizers, we trained vanilla MLP, linear IFM, and CMG using three optimizers: SGD, AdamW, and Muon. SGD with momentum served as the classical baseline optimizer. AdamW was included as a widely used adaptive optimizer with decoupled weight decay. Muon was included as a recent optimizer designed for stronger performance and improved convergence on modern architectures. For all optimizer settings, the same MLP backbone, scheduler, batch-size search, epoch number, and random seeds were used within each dataset (Table 2).

**Table 2:** Configuration of MLP training.

Parameter	Value
Optimizer	AdamW, SGD, Muon
Batch sizes	{32, 64, 128}
Initial learning rates	AdamW: {0.003, 0.001, 0.0003} SGD, Muon: {0.025, 0.01, 0.001}
Dropout rate	0.3
Learning rate scheduler	Supra-linear scheduler
Seeds	{0, 1, 2}
MLP hidden layer size	CIFAR10 [1,024, 512] CIFAR100 [2048, 1,024]

To provide a preliminary assessment beyond MLP, we also tested CMG as an IFM on a simple CNN. The CNN consisted of three convolutional blocks with output channels [32, 64, 128] followed by adaptive global average pooling and a final linear classifier. We train the simple CNN on both CIFAR10 and CIFAR100, and Muon is adopted as the optimizer. The training hyper-parameter settings, such as scheduler, learning rate search, batch-size search, epoch number, and random seeds, are the same as MLP in Table 2.

To encourage exploration at the beginning of training, the initial parameters in CMG are sampled from uniform distributions, where *μ* values are sampled from U(0.25,0.75) and I values are sampled from U(0,1) .

### 4.4 Calculation of CMG gradients

When we train the CMG modulator during backpropagation, for logistic-phase CMG, the gradients for x, *μ*, and I can be easily calculated from the mathematical expression, but for logit-phase, it needs some tweaks to calculate the approximate gradient because there lacks explicit expression for this phase. Given f marking the expression of the logistic-phase CMG.

The CMG logistic-phase meets the following conditions:

- bijective (one-to-one correspondence)
- differentiable everywhere.

Thus, we can utilize the inverse function rule (Marsden and Weinstein, 1981) to calculate the approximate gradient of x in the logit phase, which is


$${\left[f^{-1}\right]}^{\prime }(x)=\frac{1}{f^{\prime }(f^{-1}(x))}$$

where f’ can be easily derived, and the logit-phase value f^−1^(x) can be obtained from the approximation algorithm.

For the approximate gradient of *μ* in the logit phase, letting y = *f*(x, μ, I) and *F*(x, μ, I, y) = *f*(x, μ, I)-y = 0, so *F* meets the following conditions:

- There exists at least one point satisfying *F*(x, μ, I, y) = *f*(x, μ, I)-y = 0, which comes naturally given by our definition,
- Continuously differentiable with respect to all variables: x, μ, I, y
- The derivative of *F* with respect to x does not equal 0, which is $\frac{\partial F}{\partial x}=\frac{\partial f}{\partial x}\neq 0$ (see Supplementary material 3 for the proof).

Thus, we can derive the approximate gradient of μ in the logit phase using the implicit function theorem (Krantz and Parks, 2013). Specifically, we have


$$\frac{\mathrm{dx}}{\mathrm{d\mu}}=-\frac{\frac{\partial F}{\partial \mu }}{\frac{\partial F}{\partial x}}=-\frac{\frac{\partial f}{\partial \mu }}{\frac{\partial f}{\partial x}}$$

Since the partial derivatives with respect to μ and x are easy to calculate, we can derive an explicit expression for the above equation. If we further replace x = *f*^−1^(y, μ, I) on both sides with y = *f*^−1^(x, μ, I), whose value can be calculated from an approximation algorithm, this can yield the approximate derivative of logit-phase CMG with regard to μ, which is $\frac{\mathrm{dy}}{\mathrm{d\mu}}$ , and we can also derive that of I, following the same procedure.

### 4.5 Image modulation and contrast analysis

To evaluate the effects of CMG modulation, we compare each original image to its CMG-transformed counterpart using both qualitative and quantitative metrics. We compute luma (a perceptual proxy for brightness) by converting the RGB image to grayscale using the standard BT.601 weights:


$$Y^{\prime }=0.299R^{\prime }+0.587G^{\prime }+0.114B^{\prime }$$

where $R^{\prime }$ , $G^{\prime }$ and $B^{\prime }$ are gamma-encoded sRGB channels (Guidance for operational practices in HDR television production, 2022; International Telecommunication Union, 2011).

The extent of brightness contrast is quantified as the root mean square (RMS) of luma (Peli, 1990):


$$\mathrm{RMS}\;\text{contrast}=\sqrt{\frac{1}{N}\sum_{i=1}^{N}{\left(Y_{i}^{\prime }-\bar{Y^{\prime }}\right)}^{2}}$$

where N is the number of pixels and $\bar{Y^{\prime }}$ is the mean luma of all pixels in the image. A higher RMS contrast value implies a larger difference between pixel intensities in an image.

### 4.6 Quantitative evaluation metrics

When evaluating the performance of MLPs in IFM experiments, accuracy is utilized for measuring the classification precision and area under the curve across the epochs for measuring the learning speed. The area across the epochs (AAE) is the average performance of an algorithm up to a specific epoch, calculated by dividing the cumulative sum of its accuracy by the number of epochs. Bounded between [0, 1], it indicates the algorithm’s learning speed (Zhang et al., 2024).

When quantitatively evaluating the performance of neuron segmentation, a lower CREMI score is employed. It is calculated by the geometric mean of the variation of information (VOI) and adapted rand error (ARAND) between predicted segmentation and ground truth (Arganda-Carreras et al., 2015).

To evaluate numerical stability during training, we recorded the total number of non-finite loss, gradient, and parameter events. A non-finite loss event indicates that the loss became NaN or Inf. A non-finite gradient event indicates the occurrence of NaN or Inf values in the gradients, and a non-finite parameter event indicates NaN or Inf values in the model parameters. To evaluate computational overhead, we recorded peak GPU memory consumption and training time. For fair comparison of overhead across methods, peak GPU memory and training time were measured under a fixed benchmark with batch size 64 and learning rate 0.001.

## Data Availability

Publicly available datasets were analyzed in this study. This data can be found at: CIFAR-10 & CIFAR-100: https://www.cs.toronto.edu/~kriz/cifar.html; CREMI: https://cremi.org/data/.
