# Supplementary material for: A generalized logistic-logit function and its application to multi-layer perceptron and neuron segmentation
Source: Front Artif Intell. 2026 Jun 9;9:1785867. doi: 10.3389/frai.2026.1785867 (PMC13287133; doi:10.3389/frai.2026.1785867)
Supplement: Supplementary file 1 [file Data_Sheet_1.DOCX]

Supplementary Material

# Generalized logistic and logit functions in the literature

The original formula of the generalized logistic Richards curve was given in Richards, 1959 as:

$$W^{1-m}=\{\begin{matrix} A^{1-m}\left( 1-be^{-kt} \right) & if m<1, \\ A^{1-m}\left( 1+be^{-kt} \right) & if m>1. \end{matrix}$$

Where $W$ is the dependent variable (weight, size, etc.), $A$ is the ultimate value of $W$ and $b$ affects the value of $W$ at initial time point. (Richards, 1959) $k$ is the growth rate constant, influencing the steepness of the curve, and $m$ is the shape parameter, controlling the asymmetry and inflection value of the curve.

The formula of the generalized logit function (glogit) is given in Prasetyo et al (Prasetyo et al., 2020) as

$$glogit(p)=\log(1-{(1-p^{1/\alpha})}^{-1/\lambda})$$

Where $p$ is probability, and the relative position of $\alpha$ and $\lambda$ determines the shape of the curve. (Prasetyo et al., 2020) When $\alpha=\lambda=1$, the function is reduced to standard logit function. The response function of glogit has negative skewness for $\alpha<\lambda$, positive skewness for $\alpha>\lambda$ and symmetric for $\alpha=\lambda$. When α > 1 and λ > 1, the response function has lighter tails, while if α < 1 and λ < 1 then there are heavier tails.

# Derivation of CMG

In Richards curve, the expression of $W$ can be written as:

$$W=\{\begin{matrix} A(1-be^{-kt})^{\frac{1}{1-m}} & if m<1, \\ A(1+be^{-kt})^{\frac{1}{1-m}} & if m>1. \end{matrix}$$

Here we should notice that when $m<1$, the curve intersects with x-axis when

$$t=\frac{ln\left( \frac{1}{b} \right)}{-k}$$

While in the case of $m>1$, the lower and upper asymptotes of $W$ are $0$ and $A$ respectively. Considering both asymptotes are critical to empirical applications, only the case of $m>1$ is kept, which is:

$$W=A(1+be^{-kt})^{\frac{1}{1-m}}, m>1$$

To enable: (1) a clearer interpretation of each parameter (2) exact lower and upper bounds on x and y axis, a reformulation to Richards curve is performed in the following.

1. First, we rewrite Richards curve as:

$$W=\frac{A}{(1+be^{-kt})^{\frac{1}{m-1}}},m>1$$

2. Let $\nu=m-1,\nu>0$, the equation becomes:

$$W=\frac{A}{(1+be^{-kt})^{\frac{1}{\nu}}},\nu>0$$

3. To explicitly show inflection point $T_{i}$ in the function, we substitute parameter $b$ with $b=e^{kT_{i}}$.

$$W=\frac{A}{(1+e^{-k\left( t-T_{i} \right)})^{\frac{1}{\nu}}},\nu>0$$

4. To further verify the inflection point of $W$ lies at ${t=T}_{i}$, we derive the inflection point of $W$, which is where the second-order derivative of $W$ equals 0.

$$t=\frac{T_{i}*k-log\left( \nu\right)}{k}$$

So only when $\nu=1$, inflection point is ${t=T}_{i}$. Setting $\nu=1$, the function becomes a logistic curve:

$$W=\frac{A}{1+e^{-k\left( t-T_{i} \right)}}$$

1. In need of both right and left asymptotes, they are added to the function, and annotated with $y_{R}$ and $y_{L}$ respectively.

$$W=y_{L}+\frac{y_{R}-y_{L}}{1+e^{-k\left( t-T_{i} \right)}}$$

2. Then a reannotation is done to make the parameters more interpretable. Let:

$$x=t$$

$$x_{I}=T_{i}$$

$$B=k$$

Where $x$ is the independent variable, $x_{I}$ is inflection point, and $B$ replace $k$ as the growth rate constant, determining the slope of the curve at inflection point, which is maximal growth rate

$$max growth rate=-\frac{k\left( yL-yR \right)}{4}$$

Also, B should be positive to keep $y_{L}$ and $y_{R}$ be the left and right asymptotes respectively, otherwise they exchange. Thus, the function is re-parameterized as:

$$f\left( x \right)=y_{L}+\frac{y_{R}-y_{L}}{1+e^{-B\left( x-x_{I} \right)}}$$

1. One feature of CMG is the definition of bounds for both x and y values. Given that $x\in\left[ x_{min},x_{max} \right]$, where $x_{min}$ is the lower bound of $x$ range at which $f\left( x \right)=y_{L}$, and $x_{max}$ is the upper bound of $x$ range at which $f\left( x \right)=y_{R}$, we introduce parameter $Q$ into the function:

$$f\left( x \right)=y_{L}+\frac{y_{R}-y_{L}}{1+Qe^{-B\left( x-x_{I} \right)}}$$

to ensure the following conditions:

$$\{\begin{matrix} f\left( x_{min} \right)=y_{L}, & whichrequires Q\left( x_{min} \right)=inf \\ f\left( x_{max} \right)=y_{R}, & whichrequires Q\left( x_{max} \right)=0 \end{matrix}$$

1. In order to satisfy these conditions, $Q$ is defined as:

$$Q\left( x \right)=\frac{x_{max}-x}{x-x_{min}}$$

The function now becomes:

$$f\left( x \right)=y_{L}+\frac{y_{R}-y_{L}}{1+\frac{x_{max}-x}{x-x_{min}}e^{-B\left( x-x_{I} \right)}}$$

Because of the introduction of Q, the inflection point of the function is not exactly at xI now, so we rename xI as deviate inflection point, but it still controls the asymmetry of the function.

1. To keep the shape of the curve invariant to the values of $x_{min}$ and $x_{max}$, we need to normalize the argument of the exponential function for the width of $x$ range:

$$f\left( x \right)=y_{L}+\frac{y_{R}-y_{L}}{1+\frac{x_{max}-x}{x-x_{min}}e^{-B\left( \frac{x-x_{I}}{x_{max}-x_{min}} \right)}}$$

2. Instead of specifying an explicit deviate inflection point, we here introduce deviate inflection point parameter $0\leq I\leq1$ to control at which proportion of x range the deviate inflection point occurs. This increases the ease of use and controllability. By our definition, the value of $x$ at deviate inflection point is:

$$x_{I}=x_{min}+I\cdot\left( x_{max}-x_{min} \right)$$

Correspondingly, the value of $f\left( x \right)$ at deviate inflection point $x_{I}$ is similar:

$$y_{I}=f\left( x_{I} \right)=y_{L}+\frac{y_{R}-y_{L}}{1+\frac{x_{max}-x_{I}}{x_{I}-x_{min}}}=y_{L}+\frac{y_{R}-y_{L}}{1+\frac{1-I}{I}}=y_{L}+I\cdot\left( y_{R}-y_{L} \right)$$

The function can be now be written as

$$f\left( x \right)=y_{L}+\frac{y_{R}-y_{L}}{1+\frac{x_{max}-x}{x-x_{min}}e^{-B\left( \frac{x-\left( x_{min}+I\cdot\left( x_{max}-x_{min} \right) \right)}{x_{max}-x_{min}} \right)}}$$

$$f\left( x \right)=y_{L}+\frac{y_{R}-y_{L}}{1+\frac{x_{max}-x}{x-x_{min}}e^{-B\left( \frac{x-x_{min}}{x_{max}-x_{min}}-I \right)}}$$

1. Since here we aim to develop a generalized logistic-logit function, inflection μ is introduced to replace growth-rate constant B to control not only the steepness of CMG but also type of the curve. Specifically, we define:

- When μ=0, the curve is a step function, where in the above formula B=∞, when x<xI, f(x) = yL, when x>xI, f(x) = yR. Where xl is the value of x at the inflection point I.
- When 0<μ<0.5, it is a logistic curve
- When μ=0.5, the curve becomes linear
- When 0.5<μ<1, the curve type is logit
- When μ=1, it becomes a constant function

We here replace parameter $B$ with $\frac{1}{\mu}-2$ which is

$$f\left( x \right)=y_{L}+\frac{y_{R}-y_{L}}{1+\frac{x_{max}-x}{x-x_{min}}e^{\left( 2-\frac{1}{\mu} \right)\left( \frac{x-x_{min}}{x_{max}-x_{min}}-I \right)}}$$

And then CMG function is defined as:

$$CMG\left( x \right)=\left\{ \begin{aligned} y_{L}+\frac{y_{R}-y_{L}}{1+\frac{x_{max}-x}{x-x_{min}}e^{\left( 2-\frac{1}{\mu} \right)\left( \frac{x-x_{min}}{x_{max}-x_{min}}-I \right)}}, if 0\leq\mu\leq0.5 \\ f^{-1}\left( x,1-\mu\right), if 0.5<\mu\leq1 \end{aligned} \right.$$

1. Let’s prove by each case:

- When μ=0

Since we know $x_{I}=x_{min}+I\cdot\left( x_{max}-x_{min} \right)$, CMG in this case can be reformulated as

$$f\left( x \right)=y_{L}+\frac{y_{R}-y_{L}}{1+\frac{x_{max}-x}{x-x_{min}}e^{\left( 2-\frac{1}{\mu} \right)\left( \frac{x-x_{I}}{x_{max}-x_{min}} \right)}}$$

When μ=0, the term $\left( 2-\frac{1}{\mu} \right)$ becomes $-\infty$, when x>xI, f(x)=yR; when x<xI, f(x) equals yL.

Though the function is undefined at $x=x_{I}$, we set $f\left( x_{I} \right)=\mathrm{ma}x \left( y_{L},y_{R} \right)$ to guarantee that the function is defined everywhere, thus when μ=0

$$f\left( x \right)=\left\{ \begin{matrix} y_{L}, x<x_{I} \\ y_{R}, x>x_{I} \\ \max\left( y_{L},y_{R} \right), x=x_{I} \end{matrix} \right.$$

- When 0<μ<0.5,

CMG is a logistic function that extends significantly further the Richards curve.

$$f\left( x \right)=y_{L}+\frac{y_{R}-y_{L}}{1+\frac{x_{max}-x}{x-x_{min}}e^{\left( 2-\frac{1}{\mu} \right)\left( \frac{x-x_{min}}{x_{max}-x_{min}}-I \right)}}$$

- When μ=0.5

Since when μ=0.5, CMG becomes:

$$f\left( x \right)=y_{L}+\frac{y_{R}-y_{L}}{1+\frac{x_{max}-x}{x-x_{min}}e^{\left( 0 \right)\left( \frac{x-x_{min}}{x_{max}-x_{min}}-I \right)}}=y_{L}+\left( x-x_{min} \right)\cdot\frac{y_{R}-y_{L}}{x_{max}-x_{min}}$$

Which is a linear function

- When 0.5<μ<1

To generalize the equation to the domain of logit function, we represent the logit function as the inverse function of the generalized logistic function at 1-μ. In other words, if $f\left( x,\mu\right)$ is a generalized logistic function for 0<μ<0.5, then the generalized logit function is defined for the domain 0.5<μ<1 and the equation become

$$f^{-1}\left( x,1-\mu\right)$$

Since the inverse function does not have an explicit expression, we approximate it computationally with following algorithm:


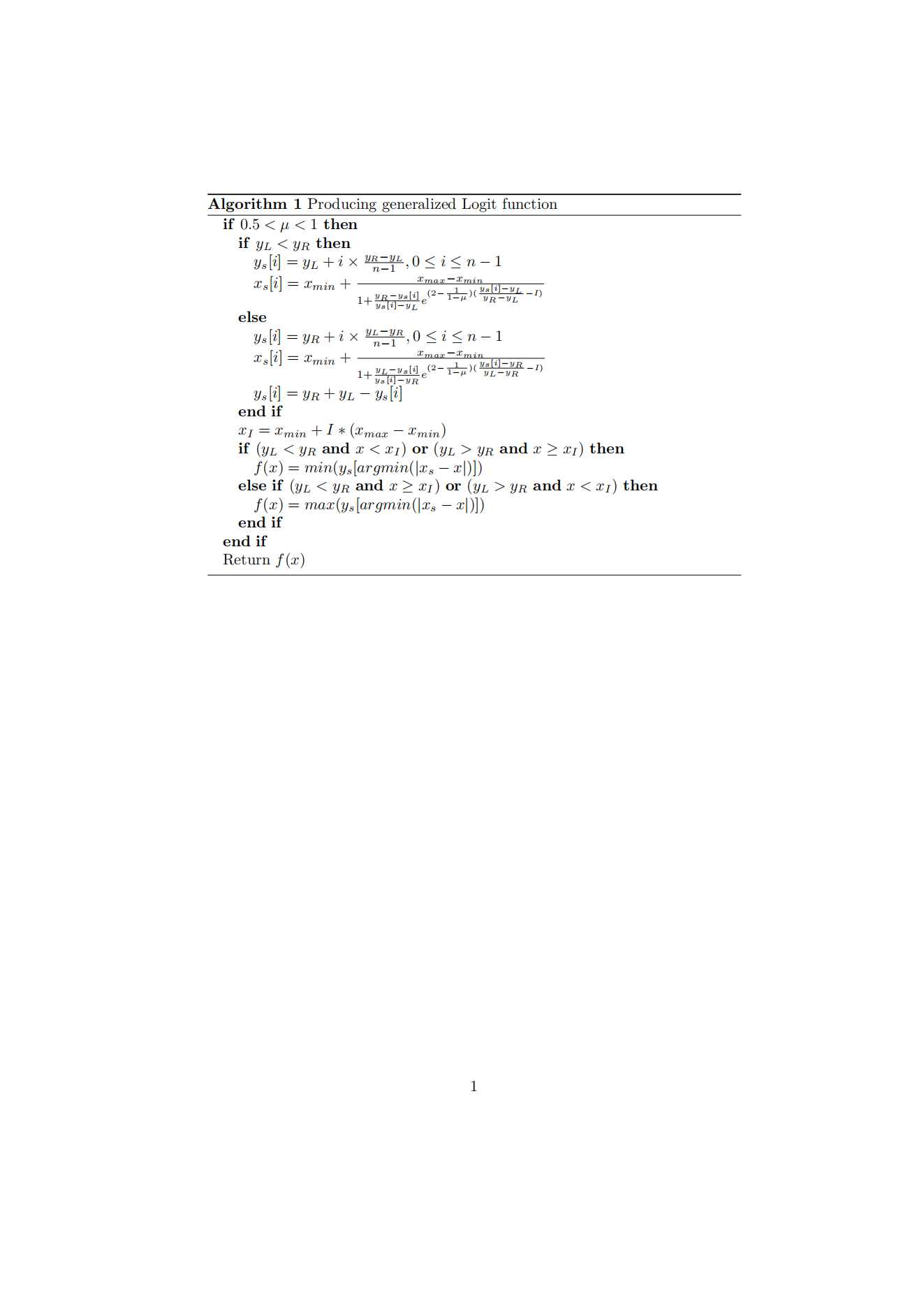


Where n is the precision parameter determining the precision of approximation.

- When μ=1

The function is the inverse of the step function, which is equivalent to a constant function with value $y_{I}$, therefore in this special case the function can be defined as:

$$f\left( x \right)=y_{I}=y_{L}+I\left( y_{R}-y_{L} \right)$$

# Derivative of CMG logistic-phase with regard to x

For the logistic-phase CMG defined for $0 < \mu< 0.5$ , $x\in\left( x_{min},x_{max} \right)$ and $y_{R}-y_{L}>0$ the derivative with respect to $x$ is strictly positive:

$$\frac{dy}{dx}>0$$

The expression for the logistic-phase CMG is:

$$y\left( x \right)=y_{L}+\frac{y_{R}-y_{L}}{1+\frac{x_{max}-x}{x-x_{min}}\cdot e^{\left( 2-\frac{1}{\mu} \right)\left( \frac{x-x_{min}}{x_{max}-x_{min}}-I \right)}}$$

To simplify analysis, we introduce a normalized variable $t$ representing the relative position of $x$:

$$t=\frac{x-x_{min}}{x_{max}-x_{min}}$$

Where $t\in\left( 0,1 \right)$. Consequently, $x$ can be expressed as:

$$x=x_{min}+t\left( x_{max}-x_{min} \right)$$

Let $\alpha=\frac{1}{\mu}-2$. Since $0 < \mu< 0.5$ it follows that $\alpha>0$.The exponential term simplifies to:

$$e^{\left( 2-\frac{1}{\mu} \right)\left( t-I \right)}=e^{-\alpha\left( t-I \right)}$$

The function can now be rewritten in terms of $t$:

$$y\left( t \right)=y_{L}+\frac{C}{1+\left( \frac{1-t}{t} \right)e^{-\alpha\left( t-I \right)}}$$

Where $C=y_{R}-y_{L}>0$.

By chain rule:

$$\frac{dy}{dx}=\frac{dy}{dt}\cdot\frac{dt}{dx}$$

First compute $\frac{dt}{dx}$:

$$\frac{dt}{dx}=\frac{1}{x_{max}-x_{min}}>0$$

Next, we prove $\frac{dy}{dt}>0$. Let:

$$D\left( t \right)=1+\left( \frac{1-t}{t} \right)e^{-\alpha\left( t-I \right)}$$

Then $y\left( t \right)=y_{L}+C\cdot\left[ D\left( t \right) \right]^{-1}$, and:

$$\frac{dy}{dt}=-C\cdot\left[ D\left( t \right) \right]^{-2}\cdot\frac{dD}{dt}$$

Since $C > 0$ and $\left[ D\left( t \right) \right]^{-2}>0$, the sign of $\frac{dy}{dt}$ depends on $-\frac{dD}{dt}$. We now show that $\frac{dD}{dt}<0$.

Let $U\left( t \right)=\frac{1-t}{t}$ and $V\left( t \right)=e^{-\alpha\left( t-I \right)}$, so $D\left( t \right)=1+U\left( t \right)V\left( t \right)$. By the product rule:

$$\frac{dD}{dt}=U^{'}\left( t \right)V\left( t \right)+U\left( t \right)V^{'}\left( t \right)$$

Compute the derivatives

$$U\left( t \right)=\frac{1-t}{t}=t^{-1}-1>0$$

$$U^{'}\left( t \right)=-t^{-2}=-\frac{1}{t^{2}}<0$$

$$V\left( t \right)=e^{-\alpha\left( t-I \right)}>0$$

$$V^{'}\left( t \right)=-\alpha e^{-\alpha\left( t-I \right)}=-\alpha V\left( t \right)<0$$

Substitute into equation:

$$\frac{dD}{dt}=\left( -\frac{1}{t^{2}} \right)V\left( t \right)+U\left( t \right)\left( -\alpha V\left( t \right) \right)$$

$$=-V\left( t \right)\left[ \frac{1}{t^{2}}+\alpha U\left( t \right) \right]$$

$$=-V\left( t \right)\left[ \frac{1}{t^{2}}+\alpha\left( \frac{1-t}{t} \right) \right]$$

Since $V\left( t \right)>0$, $\frac{1}{t^{2}}>0$, $\alpha> 0$, and $\frac{1-t}{t}>0$ for $t\in\left( 0,1 \right)$, so:

$$\frac{dD}{dt}<0$$

Since $C > 0$ and $\left[ D\left( t \right) \right]^{2}>0$,

$$\frac{dy}{dt}=-C\cdot\left[ D\left( t \right) \right]^{-2}\cdot\frac{dD}{dt}>0$$

Finally, knowing $\frac{dt}{dx}>0$

$$\frac{dy}{dx}=\frac{dy}{dt}\cdot\frac{dt}{dx}>0$$

So, for $y_{R}-y_{L}>0$, the derivative of the logistic-phase CMG with regard to $x$ is strictly positive. Similarly, it can also be proved that the derivative of the logistic-phase CMG with regard to is strictly negative for $y_{R}-y_{L}>0$.

# Comparison between trainable 2-parameter functions

To show the performance boost brought by CMG is not simply from increasing the number of parameters in the network, we also tested other learnable functions with 2 tunable parameters, a and b, as its counterpart for a fair comparison.

- Linear transformation function y = ax + b
- Cubic transformation function y = ax^3^ + b
- Adaptive Sigmoid function y = Sigmoid(ax + b) proposed in Kaseb et al(Kaseb et al., 2023)
- Adaptive Tanh function y = Tanh(ax + b) proposed in Kaseb et al(Kaseb et al., 2023)

For the learnable 2-parameter functions, $a$ values are sampled from $N\sim\left( 0,1 \right)$ and b values are sampled from $N\sim\left( -1,1 \right)$. CMG-GLLF is a monotonically increasing function as input feature modulator to represent a modulation process where stronger input signals should produce stronger responses — a property that is also true for many natural and biological systems. For a fair comparison focused on shape flexibility, all learnable functions are constrained to be also monotonically increasing as CMG-GLLF is during training.

Supplementary Figure 1 depicts the test accuracy curves of CMG and the other learnable 2-parameter functions, which shows that CMG achieves the best accuracy and AAE among the IFMs.


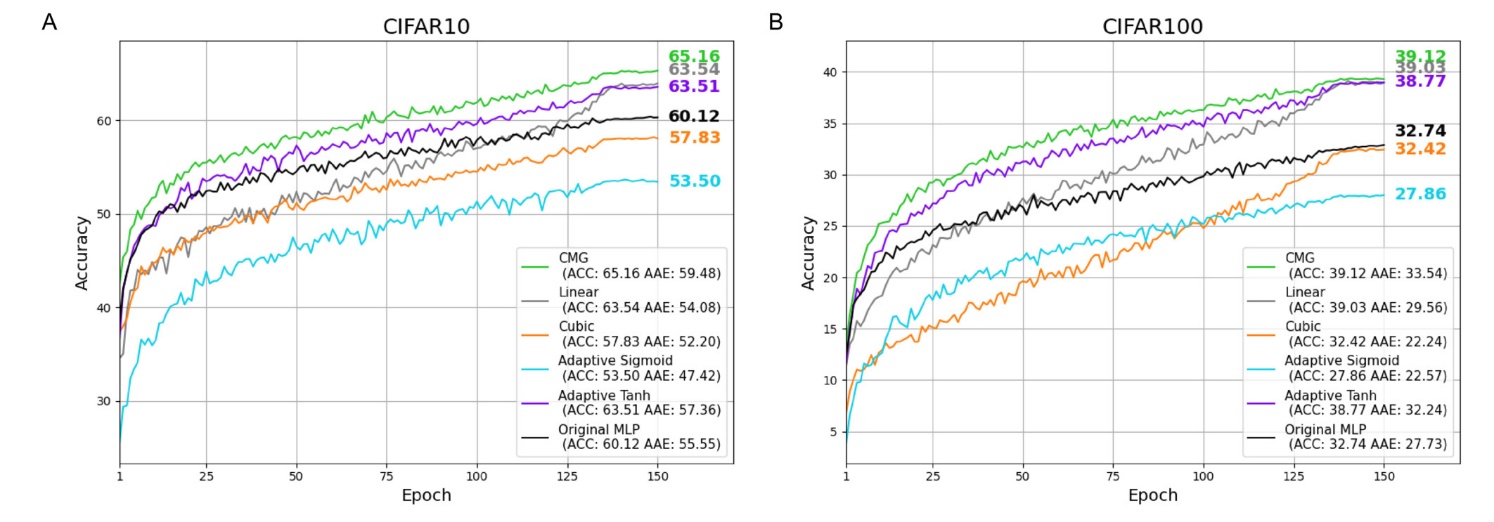


**Supplementary Figure 1.** Test accuracy curve of various learnable 2-parameter functions on CIFAR10 (A) and CIFAR100 (B) The accuracies of each trial are also shown beside the curve.

# Comparing CMG against trainable 4-parameter functions

Being curious about if CMG can outperform learnable functions with more than 2 parameters, we compare CMG against SReLU, (Jin et al., 2015) a learnable 4-parameter function, whose expression is:

$$SReLU(x)=\left\{ \begin{aligned} t^{l}+a^{l}(x-t^{l}), &for x\leq t^{l} \\ x , &for t^{l}<x<t^{r} \\ t^{r}+a^{r}(x-t^{r}), &for x\geq t^{r} \end{aligned} \right.$$

Where $t^{l}$, $t^{r}$, $a^{l}$ and $a^{r}$ are learnable during back propagation.

The initial parameters are set as: $t^{l}$ and $a^{r}$ are initialized at 0 and 1 respectively, while $a^{l}$ values are drawn from a uniform distribution $N\sim\left( 0,1 \right)$ and $t^{r}$ values drawn from $N\sim\left( 0,5 \right)$ following the empirical values employed in Keras extension library’s implementation of SReLU and Mocanu et al.(*Keras-Team/Keras-Contrib: Keras Community Contributions*, n.d.; Mocanu et al., 2018) Similar to learnable 2-parameter functions, we here keep the monoticity of SReLU. The results shown in Supplementary Figure 2 indicates that for CIFAR10, the CMG outperforms SReLU in both accuracy and AAE, while on CIFAR100 the accuracy of CMG is lower than SReLU but has a higher learning speed.


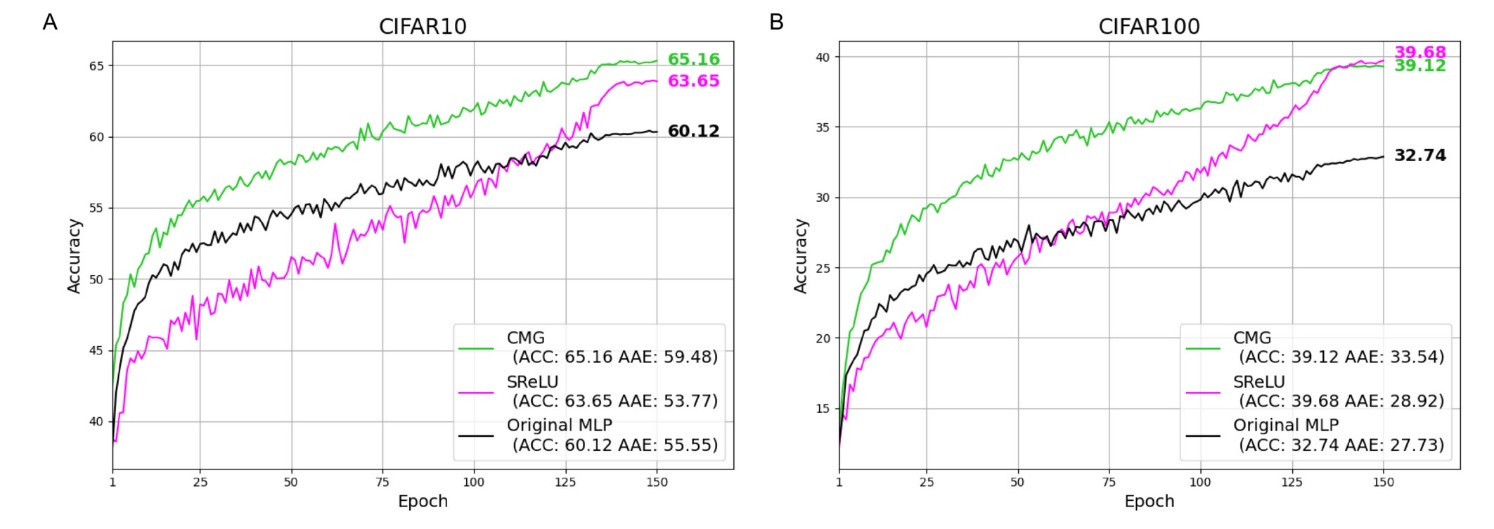


**Supplementary Figure 2.** Comparing the test accuracy curve of CMG against a 4-parameter learnable function on CIFAR10 (A) and CIFAR100(B) The accuracy of each trial is also shown beside the curve

# Numerical stability and computational overhead analysis on MLP

To verify the numerical stability of CMG on MLP trained with different optimizers, during training we measure the occurrence of events including non-finite loss (NF Loss), non-finite gradient (NF Grad) and non-finite parameters (NF Param) during training shown in Supplementary Table 1. The results show that training is stable for CMG across different optimizers. Regarding computational overhead analysis, we measure peak GPU memory in MB and training time under the condition of (batch size = 64, learning rate = 0.001). As shown in Supplementary Table 2, CMG requires far more GPU memory and training time compared to Linear IFM and vanilla MLP. This is caused by the need for using approximation algorithm to calculate the logit-phase CMG values in the forward pass.

**Supplementary Table 1.** Numerical stability analysis on MLP. The occurrence of events including non-finite loss (NF Loss), non-finite gradient (NF Grad) and non-finite parameters (NF Param) during training on MLP are recorded

| **Optimizer** | **Method** | **CIFAR10** | | | **CIFAR100** | | |
| --- | --- | --- | --- | --- | --- | --- | --- |
|  |  | **NF Loss** | **NF Grad** | **NF Param** | **NF Loss** | **NF Grad** | **NF Param** |
| SGD | Vanilla MLP | 0 | 0 | 0 | 0 | 0 | 0 |
|  | Linear | 0 | 0 | 0 | 0 | 0 | 0 |
|  | CMG | 0 | 0 | 0 | 0 | 0 | 0 |
| AdamW | Vanilla MLP | 0 | 0 | 0 | 0 | 0 | 0 |
|  | Linear | 0 | 0 | 0 | 0 | 0 | 0 |
|  | CMG | 0 | 0 | 0 | 0 | 0 | 0 |
| Muon | Vanilla MLP | 0 | 0 | 0 | 0 | 0 | 0 |
|  | Linear | 0 | 0 | 0 | 0 | 0 | 0 |
|  | CMG | 0 | 0 | 0 | 0 | 0 | 0 |

Supplementary Table 2. Computational Overhead Analysis On MLP. Peak GPU memory in MB (GPU Mem) and estimated training time in hours (Time) are recorded. For a fair comparison between the different methods, the computational overheads are measured using a fixed benchmark (batch size = 64, learning rate = 0.001)

| **Optimizer** | **Method** | **CIFAR10** | | **CIFAR100** | |
| --- | --- | --- | --- | --- | --- |
|  |  | **GPU Mem (MB)** | **Time (h)** | **GPU Mem (MB)** | **Time (h)** |
| SGD | Vanilla MLP | 122.83 | 0.49 | 194.39 | 0.51 |
|  | Linear | 122.9 | 0.53 | 194.46 | 0.55 |
|  | CMG | 1608.39 | 0.89 | 1644.67 | 0.92 |
| AdamW | Vanilla MLP | 135.86 | 0.49 | 226.79 | 0.52 |
|  | Linear | 136.95 | 0.55 | 226.88 | 0.56 |
|  | CMG | 1621.94 | 0.91 | 1677.1 | 0.93 |
| Muon | Vanilla MLP | 134.86 | 0.61 | 226.39 | 0.64 |
|  | Linear | 134.95 | 0.65 | 226.48 | 0.68 |
|  | CMG | 1608.44 | 1.04 | 1645.1 | 1.03 |

# CMG as input element modulator in CNN

We provide preliminary results on verifying CMG’s performance as input element modulator in CNN. Supplementary Figure 3 shows the test accuracy on CIFAR10 and CIFAR100 datasets. We then perform similar analysis for Simple CNN and the numerical stability results and computational overhead results are shown in Supplementary Table 3 and 4 respectively. As can be seen, CMG trained within the CNN framework has lower accuracy than vanilla CNN. In more complex structures like CNN, CMG shows evident instable training (occurrences of non-finite gradients and parameters), which does not happen to Vanilla CNN and Linear IFM. Similar to the case in MLP, CMG requires far more GPU memory and training time compared to Linear IFM and vanilla CNN.


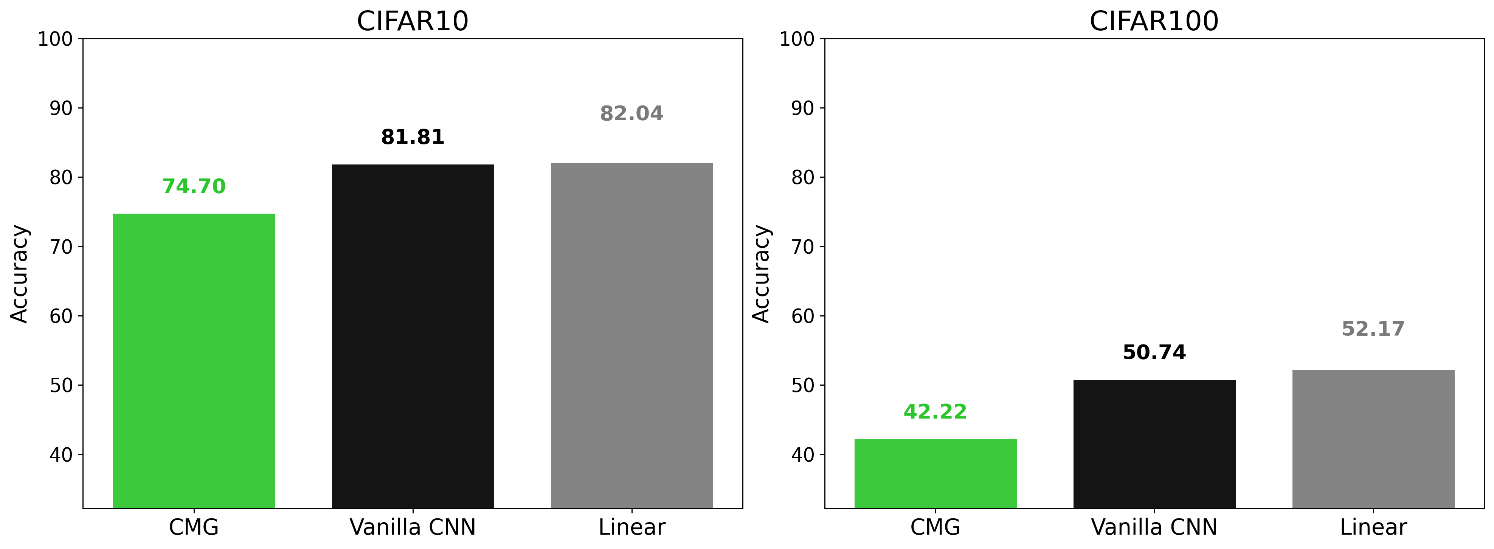


**Supplementary Figure 3.** Test accuracy comparison between CMG, vanilla CNN and linear on a simple CNN. (A) Accuracy results of different methods on CIFAR10 (B) Accuracy results of different methods on CIFAR100

**Supplementary Table 3**. Numerical stability analysis results on Simple CNN.

| **Method** | **CIFAR10** | | | **CIFAR100** | | |
| --- | --- | --- | --- | --- | --- | --- |
|  | **NF Loss** | **NF Grads** | **NF Params** | **NF Loss** | **NF Grads** | **NF Params** |
| Vanilla CNN | 0 | 0 | 0 | 0 | 0 | 0 |
| Linear | 0 | 0 | 0 | 0 | 0 | 0 |
| CMG | 0 | 27817 | 27817 | 0 | 78833 | 78833 |

Supplementary Table 4. Computational overhead analysis results on Simple CNN

| **Method** | **CIFAR10** | | **CIFAR100** | |
| --- | --- | --- | --- | --- |
|  | **GPU Mem (MB)** | **Time (h)** | **GPU Mem (MB)** | **Time (h)** |
| Vanilla CNN | 320 | 0.89 | 320 | 0.93 |
| Linear | 321 | 0.96 | 321 | 0.95 |
| CMG | 1581 | 1.27 | 1581 | 1.33 |

# Prompts provided to the Generative AI

Initial prompt: Could you please revise and refine the language of the introduction of the manuscript I provided to you

Final prompt: Please refine the language in Discussion section.

References

Jin, X., Xu, C., Feng, J., Wei, Y., Xiong, J., & Yan, S. (2015). *Deep Learning with S-shaped Rectified Linear Activation Units* (No. arXiv:1512.07030). arXiv. https://doi.org/10.48550/arXiv.1512.07030

Kaseb, Z., Xiang, Y., Palensky, P., & Vergara, P. P. (2023). Adaptive Activation Functions for Deep Learning-based Power Flow Analysis. *2023 IEEE PES Innovative Smart Grid Technologies Europe (ISGT EUROPE)*, 1–5. https://doi.org/10.1109/ISGTEUROPE56780.2023.10407913

*keras-team/keras-contrib: Keras community contributions*. (n.d.). Retrieved October 2, 2025, from https://github.com/keras-team/keras-contrib

Mocanu, D. C., Mocanu, E., Stone, P., Nguyen, P. H., Gibescu, M., & Liotta, A. (2018). Scalable training of artificial neural networks with adaptive sparse connectivity inspired by network science. *Nature Communications*, *9*(1), 2383. https://doi.org/10.1038/s41467-018-04316-3

Prasetyo, R. B., Kuswanto, H., Iriawan, N., & Ulama, B. S. S. (2020). Binomial Regression Models with a Flexible Generalized Logit Link Function. *Symmetry*, *12*(2), Article 2. https://doi.org/10.3390/sym12020221

Richards, F. J. (1959). A Flexible Growth Function for Empirical Use. *Journal of Experimental Botany*, *10*(29), 290–300.
